# Involvement of PKMζ in Stress Response and Depression

**DOI:** 10.3389/fncel.2022.907767

**Published:** 2022-05-20

**Authors:** Jianfeng Liu

**Affiliations:** Department of Pharmacology, School of Pharmacy, Nantong University, Nantong, China

**Keywords:** depression, stress, PKMζ, memory, antidepressants

## Abstract

The stress system in the brain plays a pivotal role in keeping humans and animals from harmful stimuli. However, excessive stress will cause maladaptive changes to the stress system and lead to depression. Despite the high prevalence of depression, the treatment remains limited. PKMζ, an atypical PKC isoform, has been demonstrated to play a crucial role in maintaining long-term potentiation and memory. Recent evidence shows that PKMζ is also involved in stress response and depressive-like behavior. In particular, it was demonstrated that stress that resulted in depressive-like behavior could decrease the expression of PKMζ in the prefrontal cortex, which could be reversed by antidepressants. Importantly, modulation of PKMζ expression could regulate depressive-like behaviors and the actions of antidepressants. These data suggested that PKMζ could be a molecular target for developing novel antidepressants. Here, I review the advance on the role of PKMζ in mediating stress response and its involvement in the development of depression.

## Introduction

Stress is a common life experience that we may come across almost daily. Humans and animals rely on the stress system in the brain to react and adapt to various stressful events and make responses. Appropriate responses to stress are essential for survival when facing life-threatening conditions (Godoy et al., 2018). However, if particular stress causes an overwhelming burden that a subject could bear, it results in maladaptive changes to the stress system in the brain, which then leads to or triggers the occurrence of many psychiatric disorders, such as depression, also known as major depressive disorder (de Kloet et al., 2005; Wohleb et al., 2016; Godoy et al., 2018). According to the 2020 National Survey on Drug Use and Health (NSDUH), about 6.7% of the adults in the United States age 18 and older suffer from depression. Despite the high prevalence, the treatment for depression remains limited.

The treatment strategy for depression includes pharmacological intervention, psychotherapy, and a combination of these two. Pharmacological intervention, such as selective serotonin reuptake inhibitors (SSRIs), is usually required for patients with moderate and severe symptoms (Davidson, 2010). Generally, pharmacological intervention is more acceptable and widely used, especially in countries and regions where psychotherapy is unavailable. However, current antidepressants have many limitations. Most currently available antidepressants require weeks of treatment before providing clinical benefits (Insel and Wang, 2009). Furthermore, depressive symptoms usually last for a long-term, even life-long for many patients, for whom daily treatment is generally required. In the past decades, emerging studies have investigated new systems and molecular targets that do not belong to the traditionally focused monoamine systems, such as serotoninergic and norepinephrinergic systems, in the hope of developing novel antidepressants (Lener et al., 2017; Shinohara et al., 2021). Recent evidence shows that PKMζ, an atypical PKC isoform that plays a pivotal role in the maintenance of LTP, may participate in the development of depression and might be one of the critical targets that mediate actions of antidepressants, suggesting that PKMζ might be a potential target for the treatment of depression. Here, I review the advance on the role of PKMζ in regulating brain function and its involvement in the pathology of depression.

## PKMζ Maintains Long-Term Potentiation and Stress-Related Memory

**FIGURE 1:**
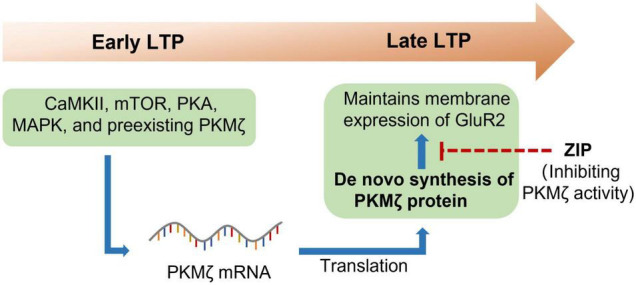
PKMζ maintains long-term potentiation. Long-term potentiation (LTP), once triggered, could last for hours or even weeks. Many protein kinases such as CaMKII, mTOR, PKA, MAPK, and preexisting PKMζ are required for the early LTP. Reactivation of these protein kinases then induced translation of PKMζ from the mRNA. The newly synthesized PKMζ then maintains the membrane expression of GluR2, which is essential for the maintenance of LTP. CaMKII, calcium/calmodulin-dependent protein kinase II; GluR2, ionotropic glutamate receptor AMPA type subunit 2; MAPK, mitogen-activated protein kinase; mTOR, mammalian target of rapamycin; PKA, protein kinase A; PKMζ, protein kinase Mζ; ZIP, ζ-inhibitory peptide.

PKMζ is an isoform of the protein kinase C (PKC), which is an enzyme that has the ability to phosphorylate serine/threonine residues (Osten et al., 1996). There are various isoforms of PKC, including conventional isoforms (α, βI, βII, and γ), novel isoforms (δ, ε, η, and θ), and atypical isoforms (ζ and ι). PKMζ is the constitutively activated form of PKCζ, which only has the ζ catalytic domain but not a regulatory domain (Hernandez et al., 2003). PKMζ was widely expressed in many brain regions, including the hippocampus, prefrontal cortex (PFC), thalamus, striatum, and so forth (Naik et al., 2000). A pioneering study by Sacktor et al. (1993) found that PKMζ was increased in the maintenance of long-term potentiation (LTP), which first linked PKMζ to the LTP. It was further shown that protein synthesis inhibitors anisomycin and cycloheximide reversed the maintenance of hippocampal LTP and prevented the increase in PKMζ (Osten et al., 1996), which suggested that PKMζ could be newly synthesized during LTP (Figure 1). The following study indicated that the *de novo* synthesis of PKMζ during LTP required many protein kinases, including phosphoinositide 3-kinase (PI3K), Ca^2+^/calmodulin-dependent protein kinase II (CaMKII), mitogen-activated protein kinase (MAPK), protein kinase A (PKA), mammalian target of rapamycin (mTOR), and preexisting PKMζ (Kelly et al., 2007). To determine the causal role of PKMζ in the maintenance of LTP, the Sacktor group synthesized the selective ζ-pseudosubstrate inhibitory peptide (ZIP) and showed that ZIP selectively prevented the maintenance of LTP without affecting baseline EPSP *in vitro* (Ling et al., 2002; Serrano et al., 2005). Thus, these data strongly suggest that PKMζ is essential for the maintenance of LTP.

Since the publication of the landmark report on hippocampal LTP by Bliss and Lomo in 1973, extensive studies have investigated this particular form of synaptic plasticity (Bliss and Lomo, 1973; Nicoll, 2017). Although many molecules had been reported to participate in LTP induction, such as CaMKII and PKA, as mentioned above, little was known about the mechanism underlying the maintenance of LTP (Lisman et al., 2012; Herring and Nicoll, 2016). Thus, when the selective involvement of PKMζ in LTP maintenance was revealed, it soon attracted much attention from many researchers, especially those who were studying the mechanism underlying learning and memory since LTP has been widely accepted as one of the primary cellular mechanisms underlying learning and memory (Lynch, 2004). In the last two decades, a large number of studies have reported the critical role of PKMζ in the storage of memory. For example, it was shown that the PKMζ inhibitor ZIP disrupted the maintenance of hippocampal LTP *in vivo* as well as abolished long-term memory in an active place avoidance task in rats (Pastalkova et al., 2006). Following studies indicated that the disruptive effects of ZIP on memory seem to be consistent across many memory tasks, including spatial memory, recognition memory, aversive and appetitive memories, which suggested that PKMζ could be a common mechanism underlying the storage of long-term memories (Pastalkova et al., 2006; Serrano et al., 2008; Migues et al., 2010).

Notably, PKMζ was demonstrated to maintain stress-related memory. This is primarily supported by numerous studies shown that PKMζ is essential for the maintenance of fear memory induced by footshock stress (Serrano et al., 2008; Kwapis et al., 2009, 2012; Migues et al., 2010; Parsons and Davis, 2011; Xue et al., 2015; Oliver et al., 2016; Schuette et al., 2016; Marcondes et al., 2021). It was shown that microinjection of the PKMζ inhibitory peptide ZIP into the basolateral amygdala (BLA) reduced the retention of cued fear memory (Serrano et al., 2008; Zhang et al., 2019), indicating that PKMζ in the BLA was a key molecule for maintaining fear memory. Consistent with this, intra-BLA injection of ZIP also disrupted the footshock-derived inhibitory avoidance memory (Serrano et al., 2008). Another study showed that the virus-mediated expression of PKMζ in the prelimbic cortex of PFC enhanced fear memory, suggesting that PKMζ in the PFC is also involved in fear memory (Xue et al., 2015). Furthermore, not only a newly formed memory but PKMζ has also been demonstrated to regulate remote fear memory (Sacco and Sacchetti, 2010). Since fear memory has been widely taken as an animal model of post-traumatic stress disorder (PTSD) (Bienvenu et al., 2021), approaches that affect the expression of PKMζ might be promising strategies to treat traumatic stress-related diseases. Nevertheless, the role of PKMζ in maintaining memory has been critically questioned in the last decade. In 2012, two independent groups reported no memory loss or LTP disruption in PKMζ knockout animals, which provided direct evidence that PKMζ might not be essential for memory or LTP (Lee et al., 2013; Volk et al., 2013). In addition, some studies showed that ZIP, which was widely used as the selective PKMζ inhibitory peptide, was not specific at all (see discussion below). Up to this point, the role of PKMζ in stress-related memory remains in debate, which needs further investigation to be fully understood.

## PKMζ Participates in Stress Response, Anxiety, and Depression

Extensive studies have demonstrated that stress, a crucial factor affecting synaptic plasticity, has dramatic influences on LTP (Peters et al., 2018). Stress could cause impairment or enhancement of LTP, which depends on various paradigms of the experienced stress, including controllability, severity, and duration (Kim et al., 2006; Abush and Akirav, 2013; Peters et al., 2018). Generally, long-lasting and uncontrollable stress is thought to impair LTP (Kim et al., 2006). Given the importance of PKMζ in LTP maintenance, it could be inferred that PKMζ might be involved in stress response. Consistently, several studies have shown that stress could affect PKMζ expression in the hippocampus and medial prefrontal cortex (mPFC), two critical brain areas that mediates stress response and depression, and PKMζ in these brain regions might meditate stress-related behaviors in some conditions. However, the related reports are controversial, and the particular role of PKMζ in mediating stress response and related disorders remain in debate.

### Effects of Stress on Hippocampal PKMζ Expression

The findings on the effects of stress on PKMζ expression in the hippocampus are mixed through the literature. One study found that in the isolated rat embryonic hippocampal neural stem cells, dexamethasone, a pharmacological treatment mimicking stress-induced glucocorticoid secretion, decreased the expression of PKMζ mRNA, and protein. This regulation was specific since dexamethasone did not affect the expression of PKCι, the other atypical PKC isoform expressed in isolated hippocampal neural stem cells (Wang et al., 2014). A recent study showed that non-human primates who experienced stress in early life showed a lifelong reduction of PKMζ in the ventral hippocampus (Fulton et al., 2021). In contrast, chronic stress enhanced the cytosolic but not synaptic expression of PKMζ in the hippocampus (Zanca et al., 2015). Consistent with these, our recent study showed that CUS caused a reduction of PKMζ in the hippocampus (Yan et al., 2018). However, the inhibitory effect of stress on PKMζ expression was inconsistent in the literature. For example, it was shown that acute stress increased the synaptic but not cytosolic expression of PKMζ in the hippocampus (Zanca et al., 2015). Single-prolonged stress (SPS), a behavioral paradigm mimicking the development of PTSD, also increased PKMζ expression in the hippocampus of rats 7 and 14 days after experiencing the stress treatment (Ji et al., 2014). Ji et al. (2014) further showed that intra-hippocampus microinjection of ZIP reduced SPS-induced depressive-like behavior in the forced swimming task and anxiety-like behavior in the open field tests and elevated plus-maze. In contrast, another study showed that the synaptic PKMζ level in the hippocampus was not altered in rats after social defeat stress, a behavioral model of depression based on social motivation (Iniguez et al., 2016). Since different types of stress were used in these studies, the discrepancy among these studies might suggest that stress type could be an essential factor determine the effects of stress on hippocampal PKMζ expression. Furthermore, it should be noted that some studies examined the cytosolic expression of PKMζ, whereas others examined the synaptic PKMζ, which might be another factor that caused the discrepancy (Iniguez et al., 2016). Therefore, these studies suggest that stress could affect hippocampal PKMζ expression, however, the effects could be influenced by many factors, such as stress paradigm and subcellular expression (cytosolic vs. synaptic).

### PKMζ in the Medial Prefrontal Cortex Is Negatively Associated With Depressive-Like Behaviors

**FIGURE 2:**
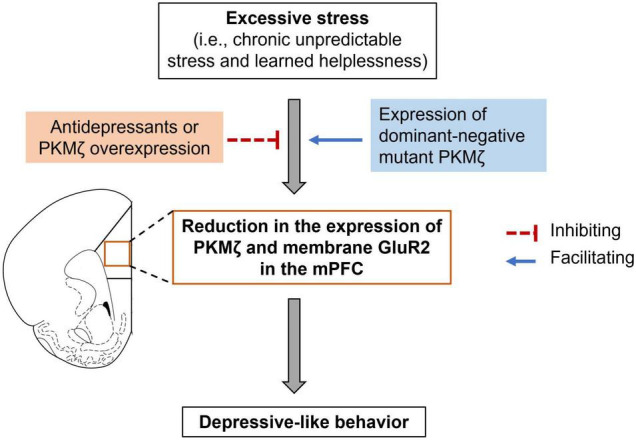
PKMζ in the mPFC participates in stress-induced depressive-like behaviors. Excessive stress, such as chronic unpredictable stress and learned helplessness, could lead to a reduction in PKMζ and membrane GluR2 expression in the medial prefrontal cortex (mPFC) as well as depressive-like behavior. Antidepressants or virus-mediated expression of PKMζ in the mPFC could relieve the depressive-like behavior. In contrast, virus-mediated expression of dominant-negative mutant PKMζ facilitated subthreshold CUS-induced depressive-like behavior.

So far, there is only one study investigated the role of PKMζ in the medial prefrontal cortex (mPFC) in mediating depressive-like behaviors (Yan et al., 2018). The study showed that PKMζ in the mPFC was decreased in two behavioral models of depression, i.e., chronic mild unpredictable stress (CUS) and learned helplessness (Figure 2; Yan et al., 2018). CUS did not change PKMζ expression in the orbitofrontal cortex, an adjacent brain region to the PFC, indicating that the PFC was the particular brain site where CUS affected PKMζ expression. Notably, SUS did not alter the expression of other PKC isoforms, including PKCα, β, θ, or λ in the PFC, suggesting that PKMζ is a unique PKC isoform influenced by CUS (Yan et al., 2018).

The causal role of PKMζ in the mPFC in depression has been implicated by studies using selective PKMζ inhibitory peptide ZIP and viruses that overexpress PKMζ or express dominant-negative mutant PKMζ (Yan et al., 2018). Intra-mPFC microinjection of ZIP enhanced stress-induced depressive-like behavior in both chronic stress and learned helplessness models (Yan et al., 2018). Because of the non-specific inhibition of ZIP on PKMζ (see details discussed below), it is hard to conclude whether PKMζ in the mPFC regulated depressive-like behaviors only based on the effects of ZIP. To confirm the role of PKMζ, Yan et al. (2018) further showed that virus-mediated expression of PKMζ in the mPFC reversed CUS- and learned helplessness-induced depressive-like behaviors as well as CUS-induced reduction in spine density and mEPCS frequency. In contrast, virus-mediated dominant-negative mutant PKMζ, which could competitively inhibit the function of endogenous PKMζ, facilitated subthreshold CUS- and learned helplessness-induced depressive-like behaviors (Figure 2; Yan et al., 2018). Unlike ZIP, virus-mediated expression of PKMζ or the dominant-negative mutant PKMζ could specifically regulate PKMζ expression or activity; thus, this study provided solid evidence that PKMζ in the mPFC mediated the development of depression.

### Antidepressants Increases PKMζ in Both the Hippocampus and Medial Prefrontal Cortex

Some evidence has shown the involvement of PKMζ in the actions of antidepressants. The selective 5-HT reuptake inhibitor fluoxetine could increase PKMζ expression and prevent dexamethasone-induced downregulation of PKMζ in isolated hippocampal neural stem cells (Wang et al., 2014). Importantly, PKMζ mediated fluoxetine-induced neurogenesis and signaling activation (Wang et al., 2014). These *in vitro* findings are consistent with our recent *in vivo* study, in which we showed that both fluoxetine and desipramine, a tricyclic antidepressant, reversed CUS-induced reduction in PKMζ expression in the mPFC. As mentioned before, antidepressants, including fluoxetine and desipramine, require several weeks of treatment to exert their antidepressant actions. Recent studies have indicated that the NMDA receptor antagonist ketamine has been shown to exert fast-acting and long-lasting antidepressant action. It has been demonstrated that ketamine could rescue chronic stress-induced molecular changes, morphological alterations of neurons, and microcircuit dysfunction in the prefrontal cortex (PFC) (Li et al., 2010, 2011; Moda-Sava et al., 2019). Intriguingly, ketamine could prevent the CUS-induced downregulation of PKMζ in the PFC; PKMζ was necessary for the antidepressant action of ketamine in the learned helplessness model. These findings insofar demonstrated that PKMζ is a critical and common target that mediates the actions of slow-acting and fast-acting antidepressants.

### PKMζ Positively Mediates Anxiety-Like Behaviors

Generally, anxiety is characterized by a persistent feeling of apprehension or dread, a specific reaction to stress. A great variety of behavioral models has been developed to mimic anxiety disorders (Kumar et al., 2013). It is not uncommon that patients with depression may also suffer from anxiety disorders (Kaiser et al., 2021). Besides fear conditioning as described above, PKMζ is also involved in other anxiety-like behaviors. Microinjection of ZIP into the hippocampus alleviated the anxiety-like behavior in rats after single prolonged stress (SPS), a paradigm used to trigger PTSD-like symptoms in animals (Ji et al., 2014). In another animal model of PSTD, it was shown that PKMζ in different brain regions exerts a time-dependent role in storing traumatic memory and mediating anxiety-like behaviors in rats exposed to predator scent stress. The study showed that injection of ZIP into the dorsal hippocampus 1 h after predator scent stress exposure disrupted anxiety-like behavior and trauma cue response 8 days later, whereas intra-insular cortex injection of ZIP 10 days after predator scent exposure showed a similar effect (Cohen et al., 2010). This suggested that PKMζ in dorsal hippocampus and insular cortex might regulate different stages of anxiety disorders. In a valproic acid model of autism, mice with valproic acid injection showed a higher level of PKMζ in the BLA. Injection of ZIP into the BLA decreased anxiety-like behavior in the VPA-injected mice (Gao et al., 2019). In another study, microinjection of ZIP into the anterior cingulate cortex reversed pain-induced anxiety-like behavior (Du et al., 2017). These studies suggested that PKMζ in many brain regions could be a common molecule that maintains different traumatic memories and mediates anxiety-like behaviors. In addition, the anxiolytic effects of ZIP are not dependent on the types of stress used to trigger anxiety-like behavior.

Consistent with the role of PKMζ in mediating stress-induced anxiety-like behaviors, PKMζ has been demonstrated to regulate the basal level of anxiety. Genetically modified mice that lack both PKCζ and PKMζ showed reduced anxiety behavior (Lee et al., 2013). In contrast, virus-mediated overexpression of PKMζ in the BLA of wild-type mice increased anxiety-like behavior (Gao et al., 2019). However, although virus-mediated overexpression of PKMζ in the prelimbic cortex enhanced fear memory, this intervention showed no effect on basal anxiety-like behavior evaluated by open field test and elevated plus-maze task (Xue et al., 2015). These studies might suggest that BLA but not the prelimbic cortex might be crucial for basal anxiety behavior.

## Potential Mechanisms of PKMζ in Regulating Depression

Evidence has illustrated the molecular mechanism underlying the role in maintaining LTP (Sacktor, 2011). In hippocampal slices, perfusion of PKMζ resulted in a robust potentiation of AMPAR-mediated excitatory postsynaptic currents (EPSCs), which could be blocked by non-NMDA glutamate receptors antagonist CNQX (Ling et al., 2002), suggesting that PKMζ was sufficient for AMPAR but not NMDAR currents. Further studies have proposed particular processes of LTP initiation and maintenance (Sacktor, 2011): (1) in the initiation of LTP, NMDA receptors are activated, which then result in the reactivation of multiple protein kinases that are essential for the removal of the translational block of PKMζ synthesis; (2) the *de novo* synthesized PKMζ is then converted into a conformation with constitutive activity after phosphorylation by phosphoinositide-dependent protein kinase 1 (PDK1); (3) the constitutively activated PKMζ increases N-ethylmaleimide-sensitive factor (NSF)/the glutamate receptor 2 (GluR2)-dependent trafficking of AMPAR and maintains the AMPAR expression at postsynaptic sites to potentiate synaptic transmission.

PKMζ was shown to be able to phosphorylate and inhibit PIN1 (protein interacting with NIMA1), a prolyl isomerase, which has the capacity for suppressing the translation of PKMζ from mRNA (Westmark et al., 2010). This self-perpetuating mechanism of PKMζ translation in synapses thus explained the maintained high levels of PKMζ and its activity, which is required for maintaining synaptic plasticity. As a critical subunit of AMPARs, GluR2 is crucial for AMPAR assembly and trafficking and determines the property of Ca^2+^ permeability and function of AMPAR (Isaac et al., 2007). It is of interest that the Ca^2+^ permeable AMPAR, which has been revealed to play an important role in short-term and long-term synaptic plasticity, contains unedited GluR2 or lacks GluR2 (Isaac et al., 2007). As described above, PKMζ regulates synaptic plasticity and LTP *via* maintaining the membrane GluR2 expression, presumably increasing the membrane expression of GluR2-containing AMPAR (Sacktor, 2011). A hypothesis could be that the GluR2 subunit composition of AMPAR switches between the initiation and maintenance of the LTP, and PKMζ might be essential for this switching (Liu and Cull-Candy, 2000); however, this requires further investigation to be determined.

Regulation of GluR2 trafficking through the interaction between PKMζ and NSF/GluR2 might also be the mechanism underlying the role of PKMζ in the PFC in depression. (Yan et al. (2018) showed that CUS and learned helplessness stress reduced PKMζ level and synaptic expression of GluR2 in the mPFC, which could be reversed by the virus-mediated expression of PKMζ. Inconsistent, virus-mediated expression of the dominant-negative PKMζ facilitated a subthreshold chronic stress-induced decrease in GluR2 in the mPFC (Ling et al., 2002). These studies may suggest that, even though different stress affects the expression of PKMζ differently in distinct brain regions, PKMζ and GluR2 levels were parallel after particular stress in a certain brain region. However, the causal role of GluR2 in mediating the function of PKMζ in stress conditions remains unclear. Elucidation of this issue may uncover the mechanism of PKMζ in response to stress and stress-related disorders.

## Considerations and Future Directions

### Selectivity of Approaches That Modulate PKMζ Activity

As described above, most work that supports the fundamental role of PKMζ in maintaining LTP and related behaviors used ZIP as a selective inhibitor of PKMζ. However, other studies have suggested that ZIP might not be an appropriate inhibitor of PKMζ.

Some evidence shows that ZIP may not be able to inhibit PKMζ. In cultured 293T cells expressing PKMζ, ZIP could not reverse PKMζ overexpression-induced increase in the phosphorylation of multiple PKC substrates. In COS-7 cells co-transfected with CKAR and PKM-RFP, ZIP did not affect the baseline normalized FRET ratio. Furthermore, ZIP did not affect MAPK2 activity in brain slices transfected with PKMζ. The authors then concluded that ZIP could not inhibit PKMζ (Wu-Zhang et al., 2012). However, the protocol used in the study could lead to a 30-fold increase in PKMζ expression, which was beyond the inhibitory ability of ZIP. Yao et al. (2013) demonstrated that ZIP was a competitive inhibitor of PKMζ and could be ineffective in inhibiting an excessively high level of PKMζ. In addition, ZIP inhibited PKMζ-induced enhancement of AMPAR potentiation but not baseline AMPAR-mediated EPSC mediated by other cellular molecules, suggesting that ZIP could selectively suppress the function of PKMζ (Yao et al., 2013).

Other studies suggested that ZIP was not selective on PKMζ. ZIP at a concentration (10 μM) could inhibit the activity of both PKCa and PKMζ (Bogard and Tavalin, 2015). ZIP also disrupted the ability of PKC to bind to AKAP79. Since AKAP79 interacts with PKCa *via* a pseudosubstrate-like mechanism, suggesting ZIP might exert its effects through the displacement of PKCa from targeted sites (Bogard and Tavalin, 2015). In a recent study, both ZIP and its control peptide scr-ZIP caused GluR1 redistribution in HEK293 cells expressing GluR1 (Bingor et al., 2020). It is of interest that HEK293 cells did not express PKMζ. The same study further showed that the effects of ZIP on AMPAR function were mediated by NOS signaling, which suggested that NOS signaling rather than PKMζ was the key target of ZIP (Bingor et al., 2020). In a physiological situation, ZIP and scr-ZIP could decrease AMPAR EPSCs in the NAc brain slices. Consistent with this, both ZIP and scr-ZIP disrupted cocaine-induced CPP, a reward memory task that requires the function of the NAc (Bingor et al., 2020).

Evidence also suggested that the effects of ZIP could be attributed to ZIP-induced cellular toxicity. One study showed that both ZIP and scr-ZIP dose-dependently caused rapid cell death of cultured hippocampal neurons (Sadeh et al., 2015). These effects might be due to ZIP and scr-ZIP-induced spontaneous activity and sustained increase in Ca^2+^ activity after application of ZIP and scr-ZIP in cultured hippocampal cells (Sadeh et al., 2015). The fact that ZIP could lead to detrimental hyperactivity in cultured hippocampal neurons suggested that ZIP was excitotoxic to neurons (Sadeh et al., 2015). In contrast, one study indicated that ZIP could lead to neural silence in the hippocampus *in vivo* (LeBlancq et al., 2016). LeBlancq et al. (2016) recorded local field potential from the CA1 subarea of the hippocampus with the infusion of ZIP directly into the recording area. Astonishingly, they found that ZIP caused a profound inhibition of LFP comparable to the magnitude of that induced by lidocaine, a sodium channel blocker. The duration of LFP inhibition maintained by ZIP was even longer than lidocaine. Although these two studies reported contradictory findings that ZIP excited or inhibited neural activity, it is possible that ZIP-induced inhibition of LFP might be a consequence of ZIP-induced excitotoxicity (Patel and Zamani, 2021).

Given these critical concerns on ZIP, i.e., the ineffectiveness in some conditions, non-specificity, and neurotoxicity, it should be cautious when interpreting the results used ZIP. In particular, ZIP should not be taken as a specific inhibitor of PKMζ. Other approaches rather than ZIP should be employed to determine the causal role of PKMζ in regulating brain functions and related behaviors. These approaches may include virus-mediated downregulation or expression of PKMζ in particular brain regions or subtype of cells. For example, previous studies have used viruses to overexpress PKMζ or the negative-dominant mutant PKMζ that could competitively inhibit PKMζ activity in particular brain regions could modulate animal behaviors (Shema et al., 2011; Xue et al., 2015; Yan et al., 2018).

### PKMζ Might Be a Maintenance Mechanism for Depression

Depression is a brain disorder characterized by persistently depressed mood and loss of interest. Patients usually need to take antidepressants to benefit from the treatment continually. Furthermore, some patients may stop benefiting from particular antidepressants after long-term treatment (Anderson, 2013). At least to some extent, these phenomena suggest that currently available antidepressants only transiently suppress the symptoms of depression but do not directly affect the maintenance of depression. Theoretically, a medicine that directly influences the maintenance of depression may permanently reverse the related maladaptive changes and cure depression. Since PKMζ has been implicated in the maintenance of synaptic plasticity and memory (Sacktor, 2011), it is presumed that stress-induced reduction in PKMζ in the PFC may result in a persistent dysfunction of this brain region. This could be a mechanism underlying the persistence of depressive-like symptoms. However, several questions should be addressed before concluding the role of PKMζ in the persistence of depressive-like behaviors. For example, it remains unknown how long the stress-induced reduction in PKMζ could last. Another interesting question would be whether PKMζ is a molecule that mediates the sustained effects of antidepressants.

Since PKMζ has been long taken as a memory molecule, it is of great curiosity to examine whether PKMζ could be a link between depression and memory problems. Patients with depression usually suffer short-term memory loss and are at risk of long-term memory loss (Dillon and Pizzagalli, 2018). As PKMζ in the mPFC is crucial for depressive-like behavior and memory maintenance. It could be presumed that depressive-like behavior-associated reduction in PKMζ expression in the PFC might underlie the memory problems found in patients with depression. Addressing this issue may shed light on the understanding of the relationship between stress-induced cognition dysfunction and the development of depression.

Furthermore, depression is a complicated disease that involves many brain regions (Nestler, 2015; Hare and Duman, 2020). As mentioned above, stress increased PKMζ expression in the hippocampus but decreased it in the mPFC, indicating that PKMζ in the hippocampus and mPFC may be involved in stress response and depression distinctively. Future studies are needed to address the role of PKMζ in different brain regions in regulating depression and the actions of antidepressants.

### Is PKMζ Involved in Stress Resilience?

Since PKMζ in the PFC was negatively associated with depression symptoms, it could be predicted that pharmacological or behavioral approaches that could elevate PKMζ expression in the mPFC might lead to stress resilience and protect subjects from experiencing detrimental consequences of stress, which thus prevents the development of stress-induced depression. Yan et al. (2018) showed that even though antidepressants prevented CUS- or learned helplessness-induced reduction in PKMζ expression in the PFC, they did not influence PKMζ expression in non-stressed animals. Virus-mediated expression of dominant-negative mutant PKMζ in the PFC did not induce depressive-like behaviors. These results may suggest that basal PKMζ activity in the PFC could not be critical for depressive-like behaviors. However, it would be interesting to examine whether the virus-medicated expression of PKMζ or its dominant-negative mutant would influence the resilience or susceptibility in responding to stress.

## Conclusion

Recent evidence shows that PKMζ is involved in stress response and depressive-like behaviors. PKMζ in the PFC could be a common molecule that mediates the actions of slow-acting and fast-acting antidepressants. However, the role of PKMζ in mediating stress response and depression remains largely unknown, which needs further investigation. Addressing this issue will determine whether PKMζ could be a therapeutic target for developing novel antidepressants.

## Author Contributions

The author confirms being the sole contributor of this work and has approved it for publication.

## Conflict of Interest

The author declares that the research was conducted in the absence of any commercial or financial relationships that could be construed as a potential conflict of interest.

## Publisher’s Note

All claims expressed in this article are solely those of the authors and do not necessarily represent those of their affiliated organizations, or those of the publisher, the editors and the reviewers. Any product that may be evaluated in this article, or claim that may be made by its manufacturer, is not guaranteed or endorsed by the publisher.
